# The association of diet, gut microbiota and colorectal cancer: what we eat may imply what we get

**DOI:** 10.1007/s13238-018-0543-6

**Published:** 2018-04-30

**Authors:** Jia Yang, Jun Yu

**Affiliations:** State Key Laboratory of Digestive Disease, Department of Medicine and Therapeutics, Li Ka Shing Institute of Health Sciences, CUHK Shenzhen Research Institute, The Chinese University of Hong Kong, Sha Tin, Hong Kong

**Keywords:** colorectal cancer, gut microbiota, fiber, protein, fat, metabolites

## Abstract

Despite the success of colonoscopy screening and recent advances in cancer treatment, colorectal cancer (CRC) still remains one of the most commonly diagnosed and deadly cancers, with a significantly increased incidence in developing countries where people are adapting to Western lifestyle. Diet has an important impact on risk of CRC. Multiple epidemiological studies have suggested that excessive animal protein and fat intake, especially red meat and processed meat, could increase the risk of developing CRC while fiber could protect against colorectal tumorigenesis. Mechanisms have been investigated by animal studies. Diet could re-shape the community structure of gut microbiota and influence its function by modulating the production of metabolites. Butyrate, one of the short-chain fatty acids (SCFAs), which act as a favorable source for colonocytes, could protect colonic epithelial cells from tumorigenesis via anti-inflammatory and antineoplastic properties through cell metabolism, microbiota homeostasis, antiproliferative, immunomodulatory and genetic/epigenetic regulation ways. In contrast, protein fermentation and bile acid deconjugation, which cause damage to colonic cells through proinflammatory and proneoplastic ways, lead to increased risk of developing CRC. In conclusion, a balanced diet with an increased abundance of fiber should be adopted to reduce the risk and prevent CRC.

## Introduction

Colorectal cancer (CRC) is the third most commonly diagnosed and deadly cancer in both men and women. In the USA, 140,250 new CRC cases will be estimated to be diagnosed along with 50,630 associated deaths in 2018 (Siegel et al., 2018). Although the CRC incidence is decreasing annually among older people benefiting from the large scale of colonoscopy screening in the USA, the incidence and mortality of CRC is still increasing in young people, especially in developing countries. 376,300 new cases were diagnosed with 191,000 associated deaths in China in 2015 (Chen et al., 2016). One of the most important factors driving the increase in CRC is the impact of an increasingly westernized lifestyle that significantly increases the prevalence of obesity and decreases physical activity in recent years in China (Goss et al., 2014; Varghese and Shin, 2014). It is consistent with the findings that nearly 90% of CRC cases are found to be sporadic, where an interplay between environmental factors and genetic susceptibility determinates the initiation and development of CRC. The study of geographic variation in CRC and its association with diet reported that over 90% of the gastrointestinal cancers were attributed to dietary habits (Doll and Peto, 1981). There are numerous epidemiology studies have identified specific dietary can either promote or protect from CRC. This indicates that the incidence of CRC could be lower via diet control and management.

**Figure 1:**
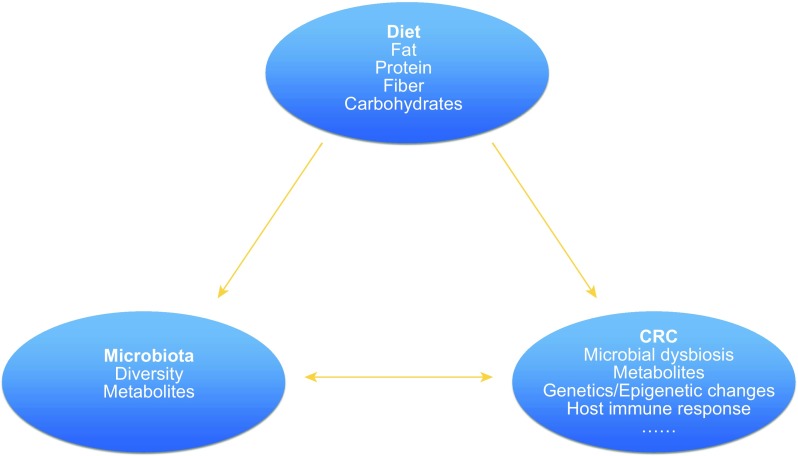
The association of diet, gut microbiota and CRC

Moreover, diet has an important impact on the composition and function of gut microbiota, which is more relevant with environmental factors rather than genetic background (Rothschild et al., 2018). Dietary fiber, fat and protein have relatively different but big effects on microbiome composition and diversity. It has been reported that short-term dietary interventions could re-shape our gut microbiome, and once return to original long-term diet, gut microbiome goes back again (Xu and Knight, 2015). Furthermore, gut microbial community structure is found to be perturbed in CRC, adenoma patients compared with healthy controls (Liang et al., 2017). Thus, this correlation strongly indicates that long-term diet could influence CRC initiation and development via gut microbiota (Fig. 1). In this review, we will provide an overview of the influence of diet on gut microbiota and CRC, the interaction between gut microbiota and CRC, and dietary intervention in CRC prevention.

## Influence of Diet on Gut Microbiota and Its Function

The human gut microbiota is a large and complex microbial community. It comprises the populations of microorganisms that live in the intestinal lumen— the bacteria, viruses, fungi, archaea, bacteriophages and protozoans. Among them, bacteria are the most abundant. In human intestine, there are 100 trillion bacteria that is ten times greater than the total number of cells in human body with at least 1,000 different species of known bacteria and carries 150 times more microbial genes than human genome (Ursell et al., 2014). The composition and function of gut microbiota varies depending on location, age, gender, race and dietary intake (Hollister et al., 2014). Recently, Rothschild et al. (2018) investigated the association between gut microbiota, environmental factors and genetic features, and demonstrated that gut microbiota community structure was predominantly shaped by environmental factors rather than genetic ancestry or individual single nucleotide polymorphisms (SNPs). Furthermore, only 1.9% of gut microbiome was estimated to be heritable and over 20% of the variance in microbiome β-diversity could be inferred from environmental factors associated with diet and lifestyle (Rothschild et al., 2018). More than that, several studies have revealed that the human gut microbiome is relatively stable in adults over time except in the case of special events such as diet intervention, infectious diarrhea or international immigration (David et al., 2014a; Faith et al., 2013). For instance, switching from a traditional African diet that is high in plant polysaccharides including fiber and low in fat and processed meat to a typical Western diet that is low in plant polysaccharides/fiber and high in fat, processed meat and sugar leads to a rapid shift in the composition and abundance of microbiome along with an increased Ki-67 index in colon tissues (Turnbaugh et al., 2008; 2009; David et al., 2014b; O’Keefe et al., 2015). These tremendous findings demonstrate that dietary intake plays an important role in shaping gut microbiota and maintaining colonic health.

**Figure 2:**
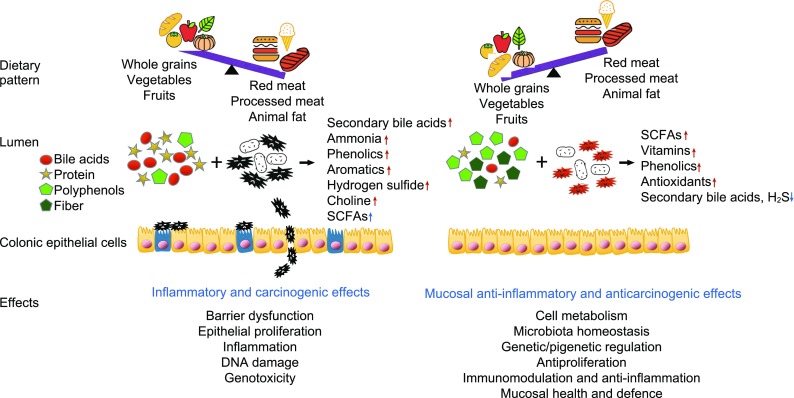
Diets influence CRC risk via gut microbiota and associated metabolites. This figure shows the effect of two different types of dietary patterns on gut microbiota and associated metabolites. Gut bacteria can promote CRC by metabolizing oncogenic dietary/digestive components such as protein and bile acids into metabolites such as secondary bile acids and hydrogen sulfide. In contrast, gut microbiota can protect against CRC by metabolizing beneficial dietary/digestive components such as plant-based polyphenols and fiber into metabolites such as butyrate. Bacteria and their metabolites can have direct effects on colonocytes via barrier dysfunction, epithelial proliferation, inflammatory, DNA damage, genotoxic ways, etc

In healthy individuals, over 90% of diet is absorbed in the small intestine and nutrients are distributed to maintain physical health. Diet residues entering the colon are mainly complex carbohydrates (fiber), along with protein residues and primary bile acids secreted by the liver in response to fat intake. These are the ones that determine the composition and function of gut microbiota and play a critical role in the maintenance of colonic health through fermentation. With a balanced diet, saccharolytic fermentation of complex carbohydrates (fiber) is predominant in producing short-chain fatty acids (SCFAs). Butyrate, as the most important member of SCFAs family, acts as a favorable source for colonocytes with mucosal anti-inflammatory and antineoplastic properties through cell metabolism, microbiota homeostasis, anti-proliferation, immunomodulatory and genetic/epigenetic regulation. In contrast, with an unbalanced Western diet, the dominant activities in colonic lumen are protein fermentation and bile acid deconjugation, which cause damage to colonic cells through proinflammatory and proneoplastic ways and thus, leads to increased risk of developing CRC (O’Keefe, 2016) (Fig. 2).

Dietary fiber is one of the determinants of microbial species diversity. One comparative study on fecal samples from vegetarians, vegans and controls who consume omnivorous diet showed a significant reduction in *Enterobacteriaceae* in vegans compared with omnivorous control subjects while vegetarians ranked between vegans and controls (Zimmer et al., 2012). A pediatric study conducted among rural African and European children revealed that African children display strikingly different gut microbial profiles compared with European children, which could be attributed to the different consumption of dietary fiber (14.2 g/d for African children and 8.4 g/d for European children) (De Filippo et al., 2010). Children from rural Africa were exclusively colonized by *Treponema* species, *Butyrivibrio*, *Prevotella* and *Xylanibacter* with increased utilization of cellulose and xylose and increased synthesis of SCFAs (De Filippo et al., 2010). Similarly, Ou et al. (2013) measured differences in gut microbiome and their metabolites in African Americans with a high risk and in rural native Africans with a low risk of developing colon cancer, and reported that the composition of microbiota was largely different with dominance of *Bacteroides* in African Americans and of *Prevotella* in natives Africans. Native Africans had more abundant butyrate producing bacteria groups with higher expression levels of microbial genes encoding for methanogenesis and hydrogen sulfide production than African Americans who were richer in expression of microbial genes encoding for secondary bile acid production. Higher levels of SCFAs were found in native African people, which was consistent with their lower risk of developing CRC (Ou et al., 2013). Furthermore, the research team performed a dietary intervention study (O’Keefe et al., 2015) of 2-week food exchanges in subjects from the same populations as described previously, in which African Americans were fed with a high-fiber, low-fat diet while rural Africans were fed with a high-fat, low-fiber Western diet. Increased butyrogenesis and saccharolytic fermentation, suppressed secondary bile acid synthesis and colonocytes proliferation were observed in the African Americans while increased secondary bile acid production and colonocytes proliferation were observed in the native Africans (O’Keefe et al., 2015). The Mechanism studies of butyrate demonstrated that butyrate could induce colonocytes apoptosis, regulate gene expression through blocking histone deacetylases (Steliou et al., 2012), and activate gluconeogenesis through cAMP-dependent mechanism (De Vadder et al., 2014). These findings further verify the protective role of fiber and promotive role of fat and protein in the process of CRC initiation and development.

When compared with complex carbohydrates fermentation, proteolytic fermentation is quantitatively smaller, which attributes to the high efficiency of protein digestion and absorption in small intestine, leading to the small amount of protein entering colon for fermentation (Silvester and Cummings, 1995). However, there are some exceptional cases in which some extreme dietary interventions like consuming Atkins or Banting diet may be adopted for body weight management. A randomized, double-blind, clinical trial aimed to manage body weight was conducted in 38 overweight individuals who received a high protein diet (HPD) for three weeks (Beaumont et al., 2017). It was reported that HPD did not alter the microbiota composition, but induced a change on bacterial metabolism with increased amino acid degradation, which was positively promoted by phylum *Firmicutes* (*Clostridiales*, *Christensenellaceae*, *Ruminococcaceae* and *Oscillospira*), and *Bacteroidetes* phylum (*Odoribacter* and *Butyricimonas*). Rectal mucosa biopsy revealed that the expression of genes playing key roles in homeostatic processes such as cell cycle or cell death was upregulated (Beaumont et al., 2017). This could be explained by the potentially toxic nitrogenous and sulfur metabolites released during protein fermentation, for instance, ammonia, nitrites, amines, hydrogen sulfide and nitrates (Windey et al., 2012).

Fat influences gut microbiota composition mainly by stimulating bile acids secretion. Microbiome is required for bile acids entering the colon to be digested into secondary bile acids. Several animal studies have shown some related evidence. Taira et al. (2015) reported that switching from a low-fat diet to a high fat diet in rats altered the microbiota composition with an increase in *Firmicutes* and decrease in *Bacteriodetes*. Similarly, another animal study (Higashimura et al., 2016) reported that microbiota could be changed when fed with a high fat diet, resulting a decrease in *Lactobacillales* and an increase in the *Clostridium* subcluster XIVa. However, the evidence for direct effect of fat on human gut microbiota is still lacking due to the mixture of meat and fat with decreased fiber in human diet. Nevertheless, substantial experimental evidence has suggested that the carcinogenic potential of secondary bile acids to colon could be potentiated by colonic butyrate deficiency (Bernstein et al., 2011; Winter et al., 2011; Toden et al., 2006, 2007; Hylla et al., 1998; Alberts et al., 1996; De Boever et al., 2000). This indicates the importance of a balanced diet for the health of the colon.

Taking all together, human dietary observational studies, dietary intervention studies and mechanism studies support the hypothesis that the risk of CRC initiation and development is influenced by the balance between microbial production of health-beneficial metabolites such as butyrate and potentially tumorigenic metabolites such as secondary bile acids.

## Influence of Diet on Colorectal Cancer

### Fiber

We usually get our daily fiber from fruit, vegetable and grain cereals. Dating back to 1970s, Burkitt who worked in Africa in 1950s suggested that high fiber diet protected from CRC based on his note of the low incidence of CRC among African people who consumed large amount of high fiber food (Burkitt, 1971). This was confirmed by other studies (Nomura et al., 2007; McCullough et al., 2003a). In 1989, Heilbrun et al. (1989) found a significant negative association of dietary fiber and colon cancer risk among low fat intake men. In this subgroup, the men consuming <7.5 g/d of dietary fiber had an adjusted relative risk for colon cancer of 2.28 (95% CI 0.93–5.60), compared to those consuming ≥14.8 g/d of dietary fiber. Steinmetz et al. (1994) also observed the inverse associations of intakes of all vegetables and dietary fiber, and colon cancer in a prospective cohort study of 41,837 women aged 55–69. In 2003, another observational EPIC study (Bingham et al., 2003) discovered that dietary fiber in foods was inversely related to incidence of large bowel cancer (adjusted relative risk 0.75 [95% CI 0.59–0.95] for the highest versus lowest quintile of intake), the protective effect being greatest for the left colon, and least for the rectum. However, no significant difference among various fiber sources on protection against CRC was observed. In 2011, with longer follow-up of EPIC study, the protective effect of dietary fiber was strengthened, and indicated that total dietary fiber was still inversely associated with colorectal cancer after a mean follow-up of 11 years (Dahm, 2011). In another large prospective cohort study that consisted of 291,988 men and 197,623 women (Schatzkin et al., 2007), although the protective effect of total dietary fiber intake was not found, whole-grain consumption was associated with a modest reduced risk of developing CRC. In a nested case-control study using food diaries, Dahm et al. (2010) discovered that intakes of absolute fiber and fiber intake density were significantly inversely associated with the risk of colorectal cancer in both age-adjusted models and multivariable models that adjusted for age, anthropomorphic and socioeconomic factors, and dietary intakes of folate, alcohol, and energy. In 2012, similar conclusion was made by a prospective Scandinavian HELGA cohort study indicating that intake of dietary fiber, especially from cereal foods, was associated with lower incidence of colon cancer (Hansen et al., 2012). The mechanisms behind these findings may include the followings. Firstly, fiber reduces concentrations of intestinal carcinogens due to the increased stool mass, increased bowel motility, and increased bacterial fermentation of resistant starch to SCFAs especially the butyrate, which keeps the health of colonocytes and enhances apoptosis and inhibits cancer cell proliferation (Burkitt, 1971; Bergman, 1990; Hamer et al., 2008). Secondly, long term fiber dominant diet may shift the gut microbiota with increased density of *Firmicutes*, which may have impact on host immune response and function as immune modulatory and anti-inflammatory effects (Fung et al., 2012; Neish, 2009).

Although there are abundant evidence supporting that fiber is beneficial to colon cells and could reduce colorectal cancer risk, there are other voices against it. Several cohort studies did not reveal the benefit of fiber in reducing CRC risk (Butler et al., 2008; Mai et al., 2003; Sanjoaquin et al., 2004; Michels et al., 2005; Otani et al., 2006; Kabat et al., 2008). In a population-based prospective cohort study conducted among Chinese women in urban Shanghai, no obvious association between fiber intake and CRC risk was observed (Shin et al., 2006). Similarly, two cohort studies conducted in Northern European population concluded that intake of dietary fiber was not associated with decreased risk of CRC (Pietinen et al., 1999; Gaard et al., 1996). Neither did the New York university women’s health study (Kato et al., 1997). The main reasons behind the discrepancy on this issue may include the followings. Firstly, there may be too many confounding factors like lifestyle, physical activity, intake of Vitamin D and so on involved in these cohort studies. These are the factors that may substantially influence the inverse association between fiber and CRC. Secondly, the intake of fiber might not reach the threshold level to produce high level of butyrate to protect against the inflammation, neoplasm and environmental carcinogens. Hence, more cohort studies with high level of fiber intake intervention are warranted in the near future.

### Fat

In 1973, Drasar and Irving found that the cancer of colon was highly correlated with fat (Drasar and Irving, 1973). This was the first time that the association between fat intake and CRC was established. Since then, the hypothesis was tested by many investigators via epidemiological studies and animal experiments. Several animal studies indicated that high fat diet could stimulate bile acids secretion and cause the regression of epithelium, damage colonic mucosa, and increase the risk of developing CRC (Chomchai et al., 1974; Narisawa et al., 1974; Rijnkels et al., 1997). However, human prospective cohort studies (Pietinen et al., 1999; Gaard et al., 1996; Kato et al., 1997; Stemmermann et al., 1984; Giovannucci et al., 1994; Bostick et al., 1994; Goldbohm et al., 1994; Chyou et al., 1996; Terry et al., 2001; Jarvinen et al., 2001b; Flood et al., 2003; Brink et al., 2004; Lin et al., 2004; Oba et al., 2006; Weijenberg et al., 2007; Butler et al., 2009) did not reveal the equivalent findings except for one study (Willett et al., 1990). In 1990, a conclusion was obtained from the nurses’ health study that a high intake of fat especially animal saturated and monounsaturated fat rather than vegetable fat, linoleic acid or cholesterol, increased the risk of developing colon cancer (Willett et al., 1990). In contrast, a significantly negative association between colon cancer and the intake of saturated fat, whether assessed on the basis of g per day or as a percentage of the caloric intake, was observed from a cohort study investigated 7,074 middle aged men of Japanese ancestry living in Hawaii during a 15-year follow-up (Pietinen et al., 1999). In 1994, Giovannucci et al. (1994) reported that intakes of total fat, saturated fat and animal fat were not related to risk of colon cancer. However, an elevated risk of colon cancer was associated with red meat intake (relative risk (RR), 1.71; 95% CI 1.15–2.55 between high and low quintiles; *P* = 0.005 for trend) (Giovannucci et al., 1994). Similarly, Goldbohm et al. (1994) found out that the increased risk of colon cancer was mainly contributed by processed meat consumption especially sausages rather than dietary fat intake. In 1996, other two prospective studies indicated no association between dietary fat and CRC risk was observed but with positive findings for age, alcohol intake, pack-years of cigarette smoking and sausages consumption (Gaard et al., 1996; Chyou et al., 1996). The New York University women’s study (Kato et al., 1997) also showed that there was no overall positive or inverse association of colorectal cancer risk with intakes of total or subclasses fat, whereas there was an inverse association with total protein. Neither was the intakes of total calories, carbohydrate, or dietary fiber (Kato et al., 1997). In 2001, Terry et al. (2001) examined total fat, fat types and specific fatty acids in relation to colorectal cancer risk in a population-based prospective cohort of 61,463 Swedish women. The result indicated that intake of total, saturated, monounsaturated and polyunsaturated fats were not associated with colorectal cancer at any sites, no matter proximal or distal colon. The ratio of saturated fat:polyunsaturated fat, docosahexaenoic acid (DHA)+ eicosapentaenoic acid (EPA):linoleic acid, ω-3:ω-6 fatty acids showed similar lack of association respectively (Terry et al., 2001). Similarly, Brink et al. (2004) found that the intake of total, saturated and monounsaturated fat was not significantly associated with CRC in a Netherlands cohort study. However, the difference from the Swedish women cohort was that high intake of dietary polyunsaturated fat and, specifically, linoleic acid was associated with an increased risk of *K-RAS* mutated colon cancer and it was also associated with G>A transitions and G>T or G>C transversions in the colon (Brink et al., 2004). Later, in 2007, after 7.3 years of follow-up of the Netherlands cohort study, linoleic acid, the most abundant polyunsaturated fatty acid in the diet, was still associated with increased risk of colon tumors with only a *KRAS* mutation and no additional truncating *Apc* mutation or lack of *MLH1* expression (Weijenberg et al., 2007). The association of fat intake and risk of CRC was also investigated in Asian population. In a prospective study (Butler et al., 2009) composed of 62,321 Singapore Chinese, a dose-dependent, positive association between saturated fat and localized colorectal cancer (Dukes A or B) was detected among women. Marine n-3 polyunsaturated fatty acid (PUFA) intake was associated with increased risk of advanced disease (Dukes C or D), in both men and women (Butler et al., 2009). However, inconsistent conclusion was drawn among Caucasian. Song et al. (2015, 2016) reported that high marine ω-3 PUFA intake was inversely associated with risk of colorectal cancer with high-level, but not low-level, FOXP3^+^ T-cell density, and microsatellite instability (MSI)-high CRC but not microsatellite stability (MSS) tumors in the nurses’ health study and health professionals follow-up cohort, suggesting a potential role of ω-3 PUFAs in cancer immunoprevention through modulation of regulatory T cells and DNA mismatch repair. In 2017, they reported that high marine ω-3 PUFA intake after CRC diagnosis is associated with lower risk of CRC-specific mortality, indicating that increasing consumption of marine ω-3 PUFAs after diagnosis may confer additional benefits to patients with CRC (Song et al., 2017). Thus, we could conclude that human studies on association of ω-3 PUFA and CRC risk are with inconsistent results and more studies are warranted to draw a definitive conclusion.

A meta-analysis performed in 1997 based on thirteen case-control studies showed the absence of impact of total dietary fat on the risk of CRC (Howe et al., 1997). Considering the potential recall and selection bias, prospective cohort studies would be more reliable than case-control studies to examine the influence of dietary fat on the risk of CRC. In 2011, a meta-analysis based on 13 prospective cohort studies that investigated the association between dietary fat intake and risk of CRC suggested that dietary fat intake might not be associated with the increased risk of CRC (Liu et al., 2011). Taking all the abundant evidence together, a definitive conclusion still could not be made, and thus, more evidence on association of dietary fat or specific types of fat and risk of CRC are needed to clarify the conclusion on this issue.

### Protein

On the basis of global epidemiological and scientific studies, evidence suggests that the risk of CRC is increased by meat consumption (O’Keefe, 2016). The two recent systematic reviews and meta-analysis published in 2011 analyzed available prospective studies of red meat and CRC, and suggested that a high intake of red and processed meat significantly increased the risk of CRC (Alexander et al., 2011; Chan, 2011). A meta-analysis (Chan, 2011) of observational studies on red and processed meat intake and risk of colorectal adenomas was conducted and found that the summary RR of colorectal cancer for the highest versus the lowest intake was 1.22 (95% CI= 1.11–1.34) and the RR for every 100 g/day increase was 1.14 (95% CI= 1.04–1.24). These indicated that high intake of red and processed meat was associated with significantly increased risk of CRC (Chan, 2011). Based on numerous epidemiological and animal studies, the International Agency for Research on Cancer (IARC) concluded that the evidence on the carcinogenicity of red and processed meat was sufficient to classify processed meat as “carcinogenic to humans” (Group 1) and red meat as “probably carcinogenic to humans” (Group 2A) in 2015 (Domingo and Nadal, 2016).

The possible mechanisms behind the positive findings on red, processed meat and CRC may include the followings. Firstly, mutagens/carcinogens like heterocyclic amines (HCA) and polycyclic aromatic hydrocarbons (PAH) are produced during the excessive cooking procedure. HCA was shown to be highly mutagenic both in vitro and in vivo experiments (Nagao, 1999; Okochi et al., 1999; Okonogi et al., 1997; Nagao et al., 1997; Burnouf et al., 2001). Rodents were found to develop multiple cancer lesions with alterations in genes including *Apc*, *β-actin* and *Ha-ras*, in different organs including breast, prostate, colon and liver, when fed with diet added with HCAs, which cause carcinogenic effect by producing DNA adducts through the formation of N-C bonds at guanine bases (Sugimura et al., 2004; Matsukura et al., 1981; Ohgaki et al., 1984a, b, 1986, 1987; Fujita et al., 1999; Esumi et al., 1989; Takayama et al. 1984a, b, 1985a, b; Tamano et al., 1994; Kato et al., 1988, 1989; Ito et al., 1991; Shirai et al., 1997; Ochiai et al., 2002). PAHs are metabolized to cause DNA damage through covalently binding to DNA (Phillips, 1983; Phillips and Grover, 1994). Thus, if the DNA damage is not repaired correctly, mutations will be induced and may initiate the development of CRC. However, the evidence of epidemiological studies investigating the interaction between red meat and HAC, PAH intake, and development of CRC was still inconsistent (Sinha et al., 2005a, b; Gunter et al., 2005; Tabatabaei et al., 2010; Cross et al., 2010; Ferrucci et al., 2009; Shin et al., 2007; Burnett-Hartman et al., 2011). Secondly, high consumption of heme iron in red meat may lead to the formation of carcinogenic N-nitroso compounds and lipid peroxidation, which both contribute to the development of CRC. Four large prospective studies showed that a high intake of heme iron was associated with a higher risk of CRC (Cross et al., 2010; Lee et al., 2004; Larsson et al., 2005; Ferrucci et al., 2012). However, other two studies failed to establish the association between heme intake and risk of CRC (Kabat et al., 2007; Zhang et al., 2011). It might be explained by the different concentration of heme iron in different red meats and then, the risk of CRC varies (Egeberg et al., 2013).

Despite the consensus on the association of red and processed meat and risk of CRC, no positive association has been presented regarding to various sources of protein except for red and processed meat. A systemic review and meta-analysis (Carr et al., 2016) analyzed meat subtypes and their association with CRC, and reported that pork intake had no overall association with CRC risk, neither in meta-analysis of cohort nor of case-control studies. Poultry consumption was consistently not associated with increased risk of CRC, its sub sites or its precursors (Carr et al., 2016). In a meta-analysis published in 2012, Wu et al. (2012) revealed that fish consumption was inversely associated with colorectal cancer. Furthermore, in 2014, another meta-analysis investigated the association of fish intake and risk of gastrointestinal (GI) cancers, and reduced risk was observed in CRC, esophageal cancer and hepatocellular cancers, suggesting that fish consumption might reduce total GI cancer incidence (Yu et al., 2014). In terms of dairy products, although several epidemiological studies indicate that milk intake might play a role in protecting against CRC due to the high level of calcium in dairy products, which is consistently agreed, there are still other studies reporting that no obvious association was found (Giovannucci et al., 1994; Kesse et al., 2005; Bostick et al., 1993; McCullough et al., 2003b; Lin et al., 2005; Jarvinen et al., 2001a; Kampman et al., 1994; Cho et al., 2004; Terry et al., 2002; Kearney et al., 1996). In 2014, a meta-analysis (Ralston et al., 2014) indicated an overall inverse risk of colon cancer of 0.74 (95% CI 0.60–0.91) in men who consumed non-fermented milk. No association was found of consumption of non-fermented milk and rectal cancer in men or non-fermented milk and CRC in women. No protective positive or negative association was found between intake of solid cheese or fermented milk and CRC (Ralston et al., 2014).

In summary, there is convincing evidence that red meat or processed meat increases risk of CRC. The evidence for poultry and fish is inconsistent while milk consumption may protect against CRC (Table 1).

**Table 1:** Dietary factors that influence CRC risk

Macronutrients	Source	Influence on CRC risk
Complex carbohydrates (fiber)	Whole grains/vegetables/fruits	Decreased risk
Protein	Red meat Processed meat Fish Poultry Milk	Increased risk Increased risk Probably decreased risk Inconsistent, probably no influence Probably decreased risk
Fat	Animal fat/fatty acids	Inconsistent evidence

## The Interaction of Gut Microbiota and Colorectal Cancer

The interaction of gut microbiota and CRC has been a major focus of research recently. The effect of environmental factors on CRC is mainly dependent on microbial dysbiosis. Indeed, microbial studies have revealed that the microbial composition has been perturbed in CRC and in precancerous lesions. A pathological imbalance in gut microbiota has been detected in subjects with CRC compared with healthy controls (Sanapareddy et al., 2012; Castellarin et al., 2012). In 1997, Dove et al. (1997) found that Germ-free mice developed 2-fold fewer adenomas than conventional controls in the medial small intestine. This finding established the hypothesis that gut microbiota contributes to the initiation and development of CRC in early days. In the following years, further studies have revealed that specific bacteria, including colibactin-producing *Escherichia coli*, *Bacteroids fragilis*, *Fusobacterium nucleatum* and *Providencia*, may contribute to colorectal carcinogenesis, together with a significant decrease in butyrate producing bacteria such as *Roseburia* and *Fecalibacterium* (Bultman, 2014; Burns et al., 2015; Arthur et al., 2012; Cuevas-Ramos et al., 2010; Grivennikov et al., 2012; Toprak et al., 2006; Uronis et al., 2009; Wu et al., 2009). The underlying mechanisms during the interaction between microbial dysbiosis and colorectal carcinogenesis include the promotion of inflammation, pathological bacteria adhesion and induction of tumorigenesis (Rubinstein et al., 2013; Kostic et al., 2013). Kostic et al. (2012) first observed that *Fusobacteria* were enriched in colorectal carcinomas, visualized within colorectal tumors by using FISH, and also identified in metastases from CRC. By using *Apc*^min/+^ mice model, they identified that *Fusobacterium nucleatum* significantly increased tumor multiplicity through recruitment of tumor-infiltrating myeloid cells to generate a proinflammatory microenvironment for tumor growth and progression (Kostic et al., 2013). Meanwhile, Rubinstein et al. (2013) suggested that *Fusobacterium nucleatum* adhered to and invaded the colonic epithelial cells, and promoted carcinogenesis through FadA, which could bind to E-cadherin, activate β-catenin signaling, and differentially regulate the inflammatory and oncogenic responses. Other virulence factors of *Fusobacterium nucleatum* such as Fap2, LPS and cell wall extracts have been identified and they may act as influential modulators in the evolution of normal colonic epithelial cells to tumor cells (Gholizadeh et al., 2017).

Despite the well-recognized associations between *Fusobacterium nucleatum* and CRC, other several species including *Parvimonas micra*, *Peptostreptococcus anaerobius*, *Solobacterium moorei* and enterotoxigenic *Bacteroides fragilis* (ETBF) were also significantly associated with CRC. In addition, many studies have demonstrated a link between bacterial virulence factors and colon malignancy. Tsoi et al. (2017) reported that *Peptostreptococcus anaerobius* was not only significantly enriched in stool samples from patients with CRC, but also in biopsies from CRC lesions compared with healthy controls. Furthermore, they also found that *P*. *anaerobius* increased colon dysplasia in azoxymethane (AOM) induced CRC mice model with gut microbiota depleted before gavaging *P*. *anaerobius*. *P*. *anaerobius* interacts with TLR2 and TLR4 to increase levels of reactive oxidative species, which promotes cholesterol synthesis and cell proliferation in colon cells (Tsoi et al., 2017). ETBF produces fragilysin (*B*. *fragilis* toxin, BFT), which is a toxin that activates the Wnt/β-catenin signaling pathway and NF-κB to induce excessive cell proliferation and inflammation (Sokol, 1999; Sears, 2009; Shiryaev et al., 2013). Furthermore, a recent published paper suggested that BFT could trigger a pro-tumorigenic signaling, multi-step inflammatory cascade requiring IL-17R, NF-κB and STAT3 signaling in colonic epithelial cells to trigger myeloid-cell-dependent distal colon tumorigenesis (Chung et al., 2018).

Moreover, many bacteria-derived metabolites such as butyrate and secondary bile acids have differentially effect on colorectal carcinogenesis, which have already been discussed previously. Thus, taking all evidence together, dysbiosis is closely associated with the initiation and development of CRC. It would be full of potential to lower the risk of CRC through modulating gut microbiome by dietary control or antibiotic treatment to eliminate tumor associated bacterial pathogens.

## Dietary Strategies in CRC Prevention

From the evidence discussed previously, we can get that an imbalanced diet leads to disturbance in community structure and function of the gut microbiota, with increased production of metabolites that could induce inflammation and proliferation and consequently increase the risk of CRC development. However, the good news is that experimental evidences strongly support that butyrate, which produced mainly through fermentation of complex carbohydrates (fibers), could suppress colorectal neoplasia. This indicates that increasing fiber intake or taking fiber supplement may protect against developing CRC. However, the problem is that how to determine the threshold intake of fiber. Many clinical trials have been conducted to determine whether fiber supplementation could reduce the risk of colorectal polyp recurrences. Although the majority of studies’ results are disappointing, we can still get some clues that fiber plays a protective role in CRC prevention. For example, a significantly reduced odds ratio was found in the subgroup taking highest level of high fiber beans in terms of preventing for the recurrence of advanced adenomatous poly in a polyp prevention trial (Lanza et al., 2006). Another inspiring result came from the study of the dietary shift intervention conducted among African Americans and native Americans, which suggested that switching diet from a high fat low fiber diet to a high fiber diet low fat diet not only changed the gut microbiota community but also reduced the proliferation of colonic epithelial cells (O’Keefe et al., 2015). In their study, the high fiber low fat diet was generated from a traditional African diet that contains more than 50 g fiber per day (O’Keefe et al., 2015).

Moreover, the positive association of obesity and CRC has been verified by many studies (Bardou et al., 2013; Ma et al., 2013; Dong et al., 2017). Obesity are frequently correlated with excessive fat dietary intake, thus making reducing fat intake from diet extremely important in CRC prevention. Taking all these evidence into consideration, we suggest a balanced diet with abundance of fiber to be adopted. Meanwhile, we suggest not to take too much animal protein frequently, especially through excessive cooking methods like grilling and barbecuing. Instead, fish, poultry and milk consumption are encouraged.

## Conclusion and Perspective

Evidence based on epidemiological, animal and human experimental studies has been shown to support the view that diet plays an important role in initiation and development of CRC. For instance, with solid consistent evidence shown in prospective cohort studies, fiber and milk are proven to be associated with decreased risk of CRC while red and processed meat is significantly related with increased risk of CRC. Meanwhile, dietary intervention could re-shape gut microbiota and alter the diet residues entering the colon. Thus, it makes dietary intervention and gut microbiota modulation promising strategies in the prevention of CRC. With the evidence available now, we strongly recommend a balanced diet with abundance of fiber to be adopted.

With the results from microbial studies and mechanism studies in human and animals, we believe it is full of potential to continue investigating the detailed role of dietary strategies in CRC prevention. There are still many unknowns to be solved. Firstly, CRC arises from various ways with different genetic backgrounds and environmental factors. Do dietary factors or nutrients function similarly or differentially in influencing CRC risk in terms of distinct etiologies? Secondly, since we already know that gut microbiota is one of the most important links between diet and CRC risk, what are the other host factors involved in the interaction of diet and CRC? What is the role of host immune response in dietary mediated CRC? These unsolved questions need to be answered with experimental models and advanced technology. Last but not least, guidelines for fiber intake is only based on the level to maintain cardiovascular health (Slavin, 2008; DeSalvo, 2016), and the dose it recommends is far away from the actual dose that fiber functions as a preventive method for CRC. So, this makes it urgent needs to determine the proper dose and duration for fiber supplement or dietary intervention to prevent or stop colorectal tumorigenesis. More clinical and preclinical studies are warranted to define the ideal recommendation for fiber supplement or dietary intervention to have proper function in reducing the risk of CRC.
